# Quantifying interaction with robotic toys in pre-term and full-term infants

**DOI:** 10.3389/fped.2023.1153841

**Published:** 2023-10-19

**Authors:** Collin Kather, Frances S. Shofer, Jeong Inn Park, Daniel Bogen, Samuel R. Pierce, Konrad Kording, Kathleen A. Nilan, Huayan Zhang, Laura A. Prosser, Michelle J. Johnson

**Affiliations:** ^1^ Rehabilitation Robotics Lab, Department of Physical Medicine and Rehabilitation, University of Pennsylvania, Philadelphia, PA, United States; ^2^ Department of Emergency Medicine, University of Pennsylvania, Philadelphia, PA, United States; ^3^ Department of Bioengineering, University of Pennsylvania, Philadelphia, PA, United States; ^4^ Department of Physical Therapy, The Children’s Hospital of Philadelphia, Philadelphia, PA, United States; ^5^ Division of Neonatology, The Children’s Hospital of Philadelphia, Philadelphia, PA, United States; ^6^ Department of Neonatology, Guangzhou Women’s and Children’s Medical Center, Guangzhou, China; ^7^ Department of Pediatrics, University of Pennsylvania, Philadelphia, PA, United States; ^8^ Division of Rehabilitation Medicine, The Children’s Hospital of Philadelphia, Philadelphia, PA, United States

**Keywords:** infant development, preterm birth, vision, movement, rehabilitation robotics, toy, neurodevelopment, neurorehabilitation

## Abstract

Infants born pre-term are at an increased risk for developmental, behavioral, and motor delay and subsequent disability. When these problems are detected early, clinical intervention can be effective at improving functional outcomes. Current methods of early clinical assessment are resource intensive, require extensive training, and do not always capture infants’ behavior in natural play environments. We developed the Play and Neuro Development Assessment (PANDA) Gym, an affordable, mechatronic, sensor-based play environment that can be used outside clinical settings to capture infant visual and motor behavior. Using a set of classification codes developed from the literature, we analyzed videos from 24 pre-term and full-term infants as they played with each of three robotic toys designed to elicit different types of interactions—a lion, an orangutan, and an elephant. We manually coded for frequency and duration of toy interactions such as kicking, grasping, touching, and gazing. Pre-term infants gazed at the toys with similar frequency as full-term infants, but infants born full-term physically engaged more frequently and for longer durations with the robotic toys than infants born pre-term. While we showed we could detect differences between full-term and pre-term infants, further work is needed to determine whether differences seen were primarily due to age, developmental delays, or a combination.

## 1. Introduction

Preterm birth affects nearly one in ten infants worldwide with a rate rising annually (1). Survivors of preterm birth often face myriad health obstacles, including behavioral, developmental, and motor delay and disability (2, 3). Severe disability at the preschool age is common in children of extremely preterm birth (birth at <27 weeks’ gestation). Infants born between 27 and 34 weeks often exhibit significant delays with fine motor, communication, and cognitive development when compared to their full-term counterparts (4–6). Early detection of and intervention for developmental impairment has shown clinical promise and may be beneficial in mitigating some potential functional sequelae, such as motor and cognitive delay and disability (7).

There is a need for robust, quantitative approaches to quantify early infant behavior in natural environments outside of the clinical setting, which in many cases worldwide may be resource constrained. Current methods of disability assessment are typically resource intensive, require extensive training of a healthcare professional, may not capture infants’ behavior in natural play environments, or are conducted at a point in an infants’ development that is too late for an early intervention with promising results. There is a need to develop assessment tools and techniques that are easy to use, affordable, and suitable for low-resource settings.

Research has shown merit in the use of sensor-based systems for studying infant movement ability and patterns. Cecchi et al. (2010) studied grasping actions and forces of infants aged 4–9 months through a set of instrumented toys and was able to identify a trend in manual forces development of infants’ grasping development (8). Sgandurra et al. (2012) explored an ecological approach to a set of toys placed at different areas of a biomechatronic gym in a set of infants aged 18–41 weeks. They found an increased ability in certain forms of power grasping and an early tendency to play bimanually (9). Serio et al. (2013) investigated novel sensor-based environment approaches and found usefulness in quantitative monitoring and measuring of infants’ motor development through instrumented toys (10). A literature review from Rihar et al. (2018) showed that sensor-supported systems, such as the *CareToy*, were beneficial and suitable for use in studying different motor patterns (11). Beani et al. (2020) reported preliminary success for the feasibility of at home early intervention using the *CareToy* system for high-risk infants (12). Research using the *CareToy* supports the use of robotics and other technology-based assessment and treatment systems in the analysis of infant behavior.

The Play and Neuro Development Assessment (PANDA) Gym is a low-cost, portable, assessment system that is designed to be used in a variety of high and low resource settings including the Neonatal Intensive Care Unit (NICU), home, at nursing homes, and low and middle-income countries (LMICs). It is focused on assessing infant supine play behavior alone and with toys at arm and feet. Our earlier works, Prosser et al. (13) and Chambers et al. (14) showed that features of infant movements collected with PANDA may have potential to identify infants with impairment at a young age. The *CareToy* system is the only existing similar technology to the PANDA [formally called the SmarToy Gym in Goyal et al. (15) and Chambers et al. (16)]. Although a precursor, *CareToy* is still not broadly available, and it was not designed to be affordable or meet assessment needs in low-resource settings such as those in LMICs. As a result, work on the PANDA Gym is different from *CareToy* system in that our goal is to develop a more accessible device for clinicians that also has the capabilities to be used as an assessment tool to examine infant neurodevelopmental delays.

Infants exhibit a variety of interactive behaviors when faced with an object or toy. Kicking, reaching, touching, grasping, and bimanual hand use are frequent infant behaviors observed in infants when they play with and without an object or toy (17–25). Kicking is one of the first intentional movements observed in infants. The Centers for Disease Control and Prevention (CDC) Milestone Moments expects most infants with typical development to develop kicking abilities around the second month of age (26). How infants perform these movements, as well as how they interact, or not interact, with an object may foreshadow delayed motor and cognitive function. An early study by Geerdink et al. (1996) found that while pre-term and full-term infants demonstrated similar kicking patterns of leg flexion and extension excursion, initial high kick frequencies decreased at a greater rate for full-term infants vs. pre-term infants with age (27). Heathcock et al. (2005) showed that while infants born full-term could adapt their kicking frequency in a task-specific manner at <9 months, infants born pre-term were unable to do so (28). Similarly, in an observational study, Deng et al. (2013) found that typical developing 5–7 month old infants with when supine had more unilateral leg movements (single flexion and single extension) than their peers with atypical development (29).

Reaching and grasping ability develops later than kicking ability, maturing at approximately 3–4 months of age (30). Guimarães et al. (2013) showed that preterm infants tend to develop reaching and bimanual ability later than their full-term counterparts (14). Soares et al. (2014) also found that preterm infants lagged behind full-term infants with regard to development of refined manual tasks such as targeted reaching and grasping and proximal adjustments (17). Further, Fallang et al. (2003) showed that movement ability appeared to get more impaired as infants of a preterm birth got older (18). In a study conducted by Passetti et al. (2015), sensor-based toys were used to measure, monitor, and promote manipulation capabilities through purposeful training for preterm infants. Preliminary data from this study showed differences in the grasping parameters in relation to infant age and the performed daily training (19). Bimanual movement and engagement portend the beginning of more complex cognitive understanding of physical and spatial engagement for infants in their first months of postnatal development (20). With toys, bimanual interaction has been seen to develop as early as 7 months in infants experiencing typical development (21). In an extensive review, Kimmerle et al. (2010) showed that while role-differentiated bimanual manipulation tended to develop in infancy, bimanual skills did not become finely tuned until after the one-year mark (22).

While kinematic interactions are widely researched, they are rarely coupled with cognitive engagement as can be represented through a simultaneous gaze interaction. Gaze is a well-studied component of human development that is closely tied to sensorimotor coordination and focused attention. In a study conducted by Van der Meer et al. (1995), healthy term infants and infants classified as neurologically at-risk were tested longitudinally between 5 and 8 months of age on the ability to use visual information to reach for an object moving at different speeds. They found that the onset of reaching and prospective control of gaze and hand varied considerably between the normal and at-risk groups, but the two children who poorly coordinated gaze with hand use were the only two of the at-risk group who were later diagnosed as having cerebral palsy (23). The above research suggests that gaze performance may also be a reliable marker of cognitive engagement with external stimuli, especially when coupled with a volitional movement interaction.

In this paper, we focus on gaining insight from video recordings of infant interactions with the toys. Guided by the literature review and analysis of the field, we developed a set of classifications to be used for coding infant physical and gaze behaviors with each toy used. We tested infants born full-term and pre-term and hypothesized that infants born full-term would engage more frequently and for a greater duration of time with the toys than their counterparts born preterm.

## 2. Methods

**Figure 1:**
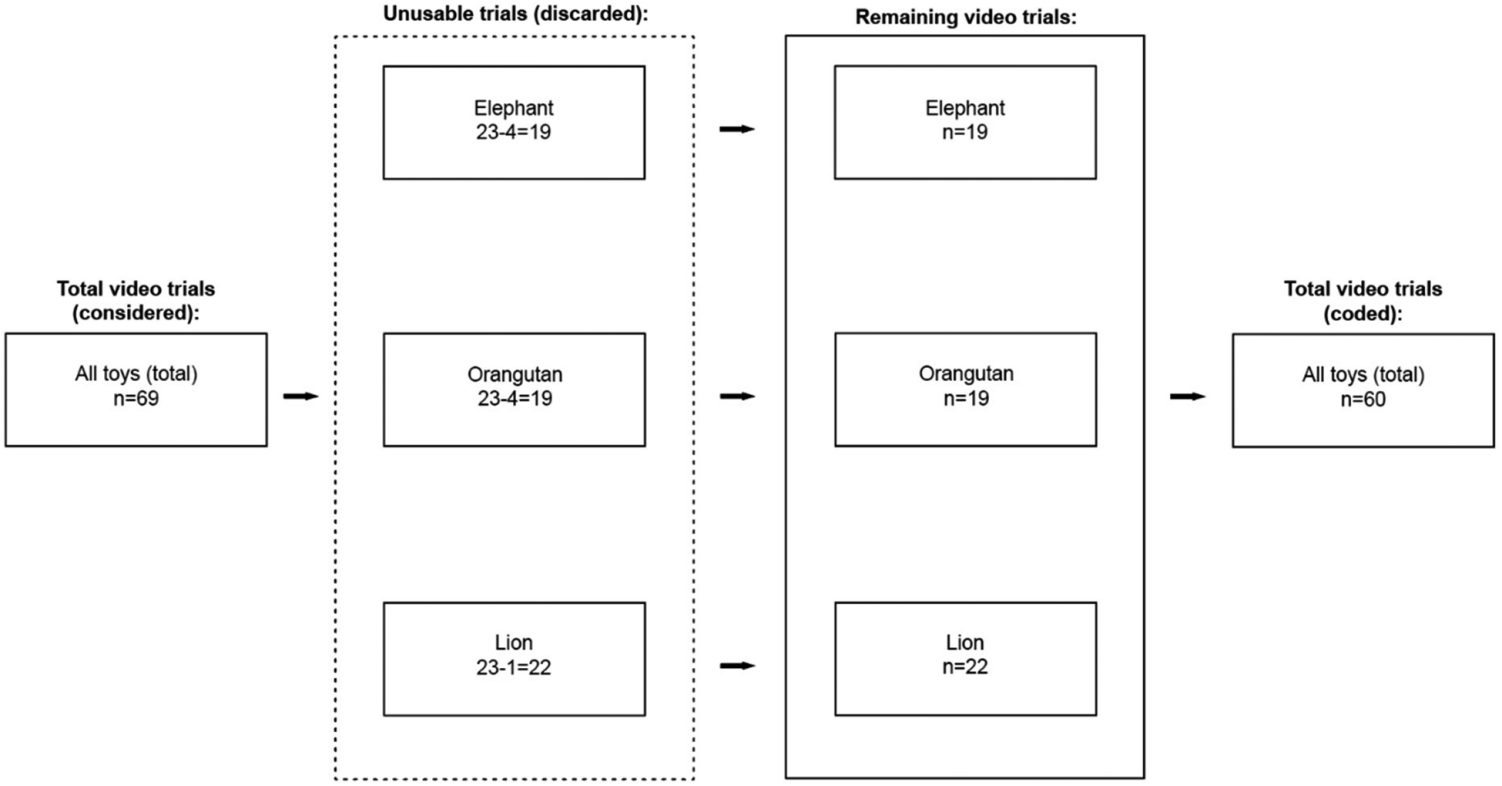
Selection of trials for analysis. Of the 34 infants in the pilot study, five were excluded due to procedural errors. Of the 29 testing sessions considered, 10 elephant videos, 11 orangutan videos, and 7 lion videos were deemed unusable and discarded from further analysis. In total, we evaluated 19 elephant trials, 18 orangutan trials, and 22 lion trials from 24 infants.

We developed a set of behavioral engagement classifications—kicking, reaching, grasping, touching—and gaze—for use in quantifying infant-toy interactions in The PANDA Gym in infants <12 months of age. We recruited and enrolled a pilot group of 34 infants of varying birth statuses to engage with the PANDA Gym in two-minute trials, of which 23 were analyzed due to technical issues with the data (see Figure 1). These infants were enrolled from October 2016 to October 2018. We coded these video segments of infant interactions with a set of three robotic toys, designed to elicit upper and lower body interactions.

### 2.1. The PANDA gym

**Figure 2:**
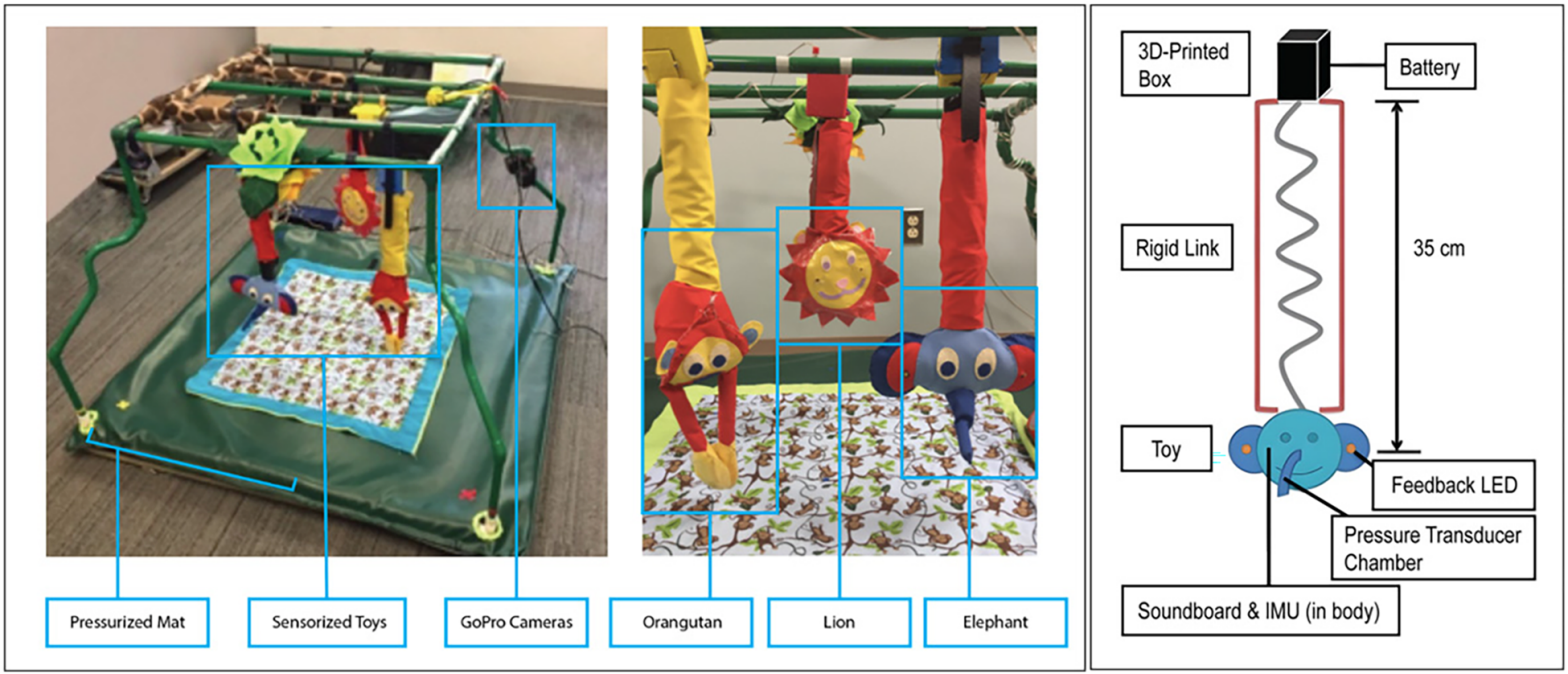
The PANDA Gym and sensor-based robotic hanging toys. The first prototype of the Gym had a 4” × 4” feet force sensing mat and two GoPro cameras with stereo views for top and side views of the infant at play. Early toy prototypes show three mechatronic toys–Orangutan, Lion, and Elephant. Each toy contains a 3-axis inertial measurement unit (IMU), force sensing resistors, custom pressure nose, LEDs and audio feedback. A sketch of the construction of the elephant toy.

The PANDA gym (Figure 2) is an instrumented play environment designed to encourage and measure natural infant interactions. The system uses a pressurized mat, sensor-based robotic toys, and 4 GoPro cameras positioned to give top view (2 cameras) and side views (2 cameras) of the interactions. The GoPro cameras were fixed in the same position with respect to the frame. The three interchangeable robotic toys—a lion, an elephant, and an orangutan—were designed for eliciting lower limb interactions, upper limb interactions, and bimanual (simultaneous use of upper limbs) interactions, respectively (15). The hanging toys composed of the 3D-printed box, rigid link, and toy itself. The box is used to hang the toys and holds the toy electronics including the batteries and a sync port to allow the toy and vision systems to be connected. The rigid link contains necessary wiring down to the toy, which contained toy-specific electronics such as Arduino boards and a custom-made PCB that is run on 3.3 V and contains an ATMEGA328P with digital pins and I2C connections for sensors. All toys were built from soft materials that were designed for use with infants. The toy outer material was removable and washable and did not appear to impact data collection.

More details on the design of our robotic toys used can be found in Goyal et al. (30) and Chambers et al. (25). They contained a 9 DOF inertial measurement unit (IMU), and touch sensors on face and ears. The elephant toy was designed to elicit upper body interactions such as grasp and touch via the long trunk and floppy ears. The elephant toy has a pressure sensor in the nose. Each time the elephant toy's trunk was grasped by the infant, an elephant noise would sound, and the ears of the toy would light up and flash. The orangutan toy was designed to elicit bimanual upper body interactions via a pair of long arms that stayed closed with a magnetic reed switch (which also allowed them to be opened and reclosed). A pleasant and engaging noise would sound if the orangutan toy's arms were opened. The lion toy was designed to elicit lower body interactions such as foot kicking and touching. A pleasant sound was played if the lion toy was kicked. Toys were tested individually and in random order in the PANDA Gym. Both the elephant and orangutan toys were placed within reach of the upper limbs during testing with the supine infants. The lion toy was placed within reach of the lower limbs during testing with the supine infants.

At the start of the experiment, infants were placed supine in the center of a pressurized mat. Five trials were completed: (1) calibration—no baby, no toy; (2) baby alone, and; (3) three toy trials in random order: (a) baby with elephant toy at arm, (b) baby with orangutan toy at arm, and (c) baby with lion toy at feet.

### 2.2. Participants

This study was approved by the Institutional Review Boards (IRBs) of the University of Pennsylvania and the Children's Hospital of Philadelphia and informed consent was obtained from parents of all infants. Infants in the pilot study were recruited from a major academic medical center, community centers and pediatric neonatal intensive care unit. Infants born pre-term and full-term (at least 36 completed weeks of gestation) between the ages of 3–11 months were eligible for participation. Infants born full-term were excluded if they had a history of any neurologic, orthopedic, cardiac, or genetic condition. Infants born preterm were born after 24–35 weeks of gestation.

Thirty-four infants enrolled in the study. Data from five were discarded for procedural inconsistencies. Several more were excluded from analysis because the infants were either crying or crawling/rolling off the gym.

Final developmental status was classified as “typically” developing or “atypically” developing from medical record review at 18 months of age for infants born preterm and from further communication with parents, when possible, for infants born full-term. Participants were considered to have atypical development if any neuromotor impairments were reported in physician examinations (i.e., hypertonicity) or if significant delays (>2 SDs below mean) in motor development were reported by developmental testing.

### 2.3. Data acquisition

Infants were placed supine on the sensor-based mat and presented with each of the three robotic toys hung above them (plus a “no toy” condition) in a randomized order. Each condition was two minutes in duration. Infants were prompted to engage with the toys through built-in sounds and lights, as described above, and their interactions were recorded on video for two minutes using the GoPro cameras. Some pre-term infants who were recruited from the NICU required supplemental oxygen or artificial ventilation at the time of testing. Any life-sustaining equipment was arranged so that it did not disturb the infants’ ability to engage with the toys.

### 2.4. Interaction classifications

**Figure 3:**
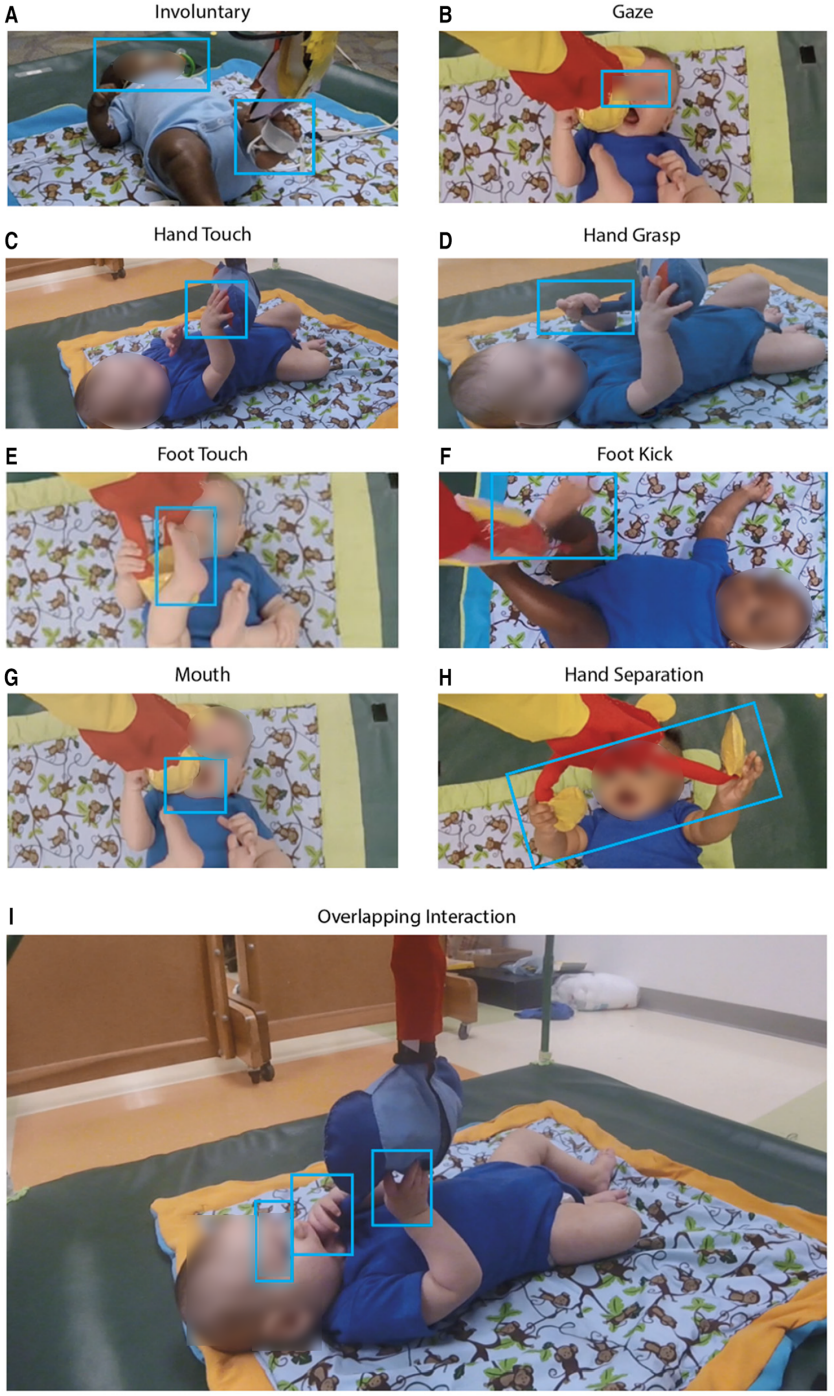
Photographic examples of infant interactions with the three toys. (**A**) Involuntary: the infant’s gaze is not focused on the elephant toy but contacts it involuntarily with a foot. (**B**) Gaze: the infant is visually focused on the orangutan toy. (**C**) Hand touch, and (**D**) hand grasp: hand grasp involved a power grasp while hand touch involves a palmar contact; distinction was also made based on shape and angle of palm and fingers around the toy. Photos (**E, F**) are examples of a foot touch being a simple contact and foot kick a forceful one, respectively. In photo (**E**) the infant puts the orangutan toy in their mouth. Photo (**H**) shows an infant separating the hands of orangutan toy which had a reed-switch keeping the arms closed. In photo (**I**) the infant simultaneously touches, grasps, gazes at, and places the toy in its mouth. Note that all other photos in Figure 5 also represent overlapping interactions.

The interaction classifications we developed describe upper body interactions, lower body interactions, mouth usage, eye gaze, and involuntary interactions with the toys (Table 1 and Figure 3). The classifications used in Table 1 were categorized as voluntary or involuntary, and further distinguished according to body part and type of interaction.

**Table 1:** Movement interactions code labels and descriptions.

Movement classification	Interaction descriptions
Involuntary	Unintentional contact with toy; toy does not appear to be the direct target of contact
Gaze	Direct eye contact with toy; determined by pupil direction and head angle
Mouth	Toy touches lips or enters mouth
L/R hand touch	Physical contact with toy but fingers do not close around
L/R hand grasp	Physical contact with toy where at least 3 fingers cup/close around
L/R foot touch	Contact of foot with toy
L/R foot kick	Contact with toy involving greater force than a touch
Hand separation	Hands of orangutan toy are separated by infant

These classifications were developed from clinical movement definitions for observed motor behaviors and literature review of infant development in the use of arms, legs, eyes, and body. Infants begin life with little volitional movement ability. Reflexive motor interaction is present from the first few weeks and becomes more purposeful at the age of three to four months. Around this time, infants can be expected to begin exploring their surroundings with their feet and kicking near objects. At around five to six months, infants should be able to reach, touch, and grasp objects. Color vision and general basic vision are present from birth, but purposeful gazing sophisticates around four to six months of age (24). Spontaneous movement ability arises around three months of age and is indicative of cyclical fluctuations, which tend to stop occurring around one year of age (25).

Between the ages of birth and 4 months, infants experiencing typical development begin exhibiting an ability to see clearly, move/rotate their head, focus their eyes, wrap their fingers around a near object, smoothly move their legs, and reach for toys. Between the ages of 4 to 8 months, they should begin to focus their gaze on, and reach and grasp for, an intended target (16). Research has also shown that full-term infants tend to interact with a desired object using their feet at least several weeks prior to using their hands in the same manner (26). Those same studies state that an infant experiencing typical development can be expected to engage with their arms and hands sooner than infants experiencing atypical development. It has been further studied that these interactions will become more fine-tuned and volitional as an infant grows and develops during the first months of postnatal life (27). Prior research has shown that it may be useful to analyze the motor development of infants in unstructured environments to detect early signs of atypical interactions and to establish standards for infantile neurodevelopmental disorder (28).

Upper body classifications defined were “touch” and “grasp,” with shape and placement of hand determining the difference between each. The lower body classifications were “touch” and “kick,” with force, speed, and intent determining the difference between each. A “mouth” interaction occurred whenever an infant placed a piece of the toy in their mouth. A “gaze” interaction occurred when the infants’ eyes locked on the toy with intent, suggesting interest; “gaze” was also used to augment the intent of any other movement classification. “Involuntary interaction” occurred when the infant physically interacted with the toy through no intent of their own. Of the voluntary interactions, upper body movements were classified as touch and grasp. “Touch” was defined as contact of the infant's hand with the toy without closing around. “Grasp” interactions were either palmar or tripod and were determined by angle of hand and positioning of fingers. For lower body interactions, movements were classified as either foot “touch” or “kick,” with relative intensity and speed being the difference between the two. Other interactions included “gaze,”—when an infant's eyes/attention locked with the toy—and “mouth,”—where an infant's mouth came into direct contact with the toy, often to bite on some piece of it.

### 2.5. Video coding process

**Figure 4:**
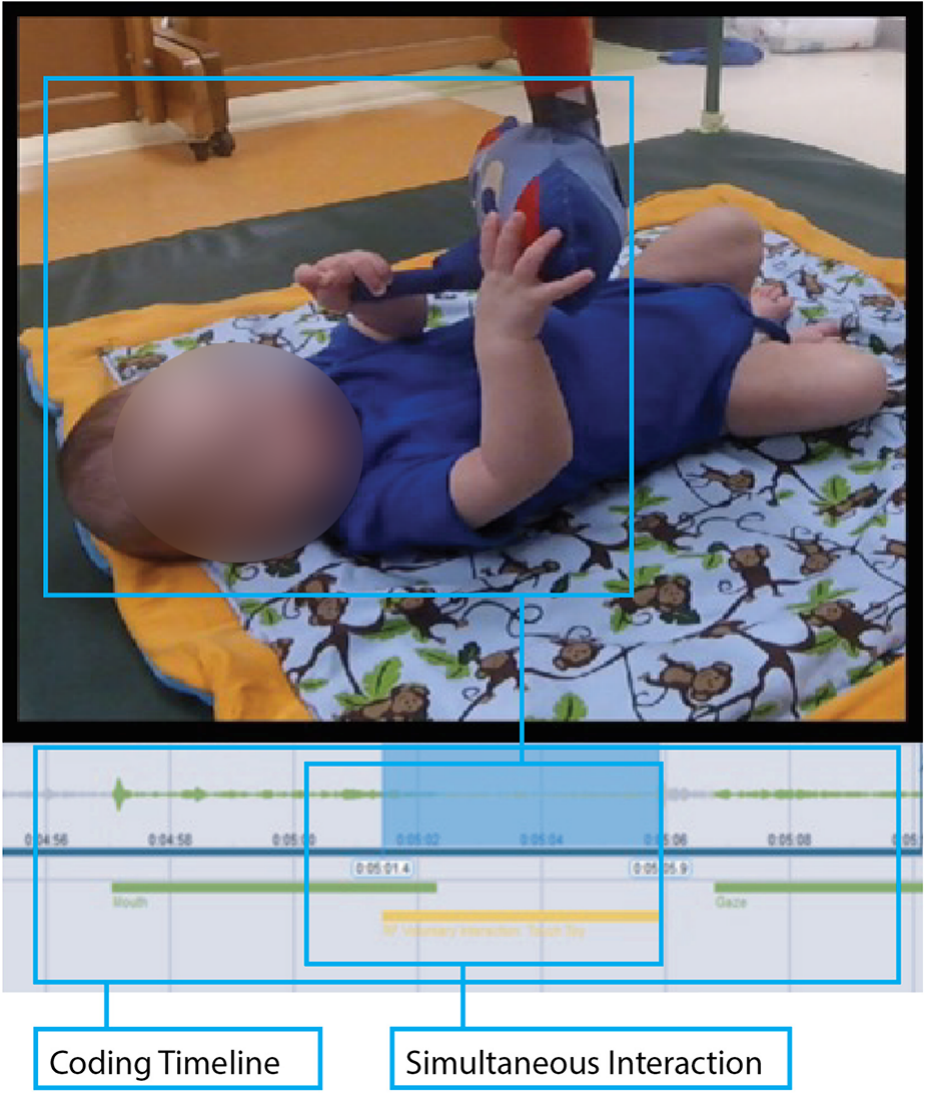
Photographic example of the video coding process. The infant is seen lying supine in the PANDA Gym with a two-minute video segment for analysis. MAXQDA software allowed the coder to assign relevant movement codes to appropriate areas along the video’s timeline. The figure above shows a simultaneous interaction and how that was coded for this infant.

Using MAXQDA video coding software (VERBI Software, 2019), infant toy interactions were assigned the relevant interaction codes synchronized to the video timeline. Figure 4 is a labeled example of the video coding process, with the codes on the timeline corresponding to the infant's interactions. Infants were coded in a randomized order and health outcome was not known to the research coder at the time of coding.

Individual interactions were initially classified as involuntary, voluntary, or gaze—with voluntary interactions further classified by type. Voluntary interactions were selected when an infant's interaction occurred with clear intent, often accompanied by a gaze interaction to confirm said intent. Voluntary interactions were coded to start when the infant contacted the toy and coded to end when the infant ceased physical contact with the toy. Involuntary interactions occurred when the infant contacted the toy without clear intent, such as when the infant was gazing outside the gym and/or flailing their limbs indiscriminately.

Due to the nature of these movement interactions, codes often overlapped, so simultaneous interactions were expected and frequent. For example, if an infant were to grab the toy while simultaneously putting its mouth around it, this would be coded as *both* a “Grasp” and “Mouth” interaction. Further, while the entire library of codes was available for each video trial, each toy was designed to elicit different behaviors, causing some codes to be used more heavily with some toys than with other toys.

To determine code reliability, two separate researchers coded a set of four videos independently. Interrater reliability was assessed using intraclass correlation coefficients and calculated as 0.978 and 0.990 for the elephant and lion toys, respectively. The remaining sample of infants was then coded by one researcher.

### 2.6. Data processing and analysis

Frequency and duration of each interaction were calculated for each trial. The data were not normally distributed, so we reported medians and interquartile ranges (IQR) for both frequency and duration measures. To compare pre-term and full-term infants, on each toy/movement, the Wilcoxon rank sum test was used due to the non-normal distributions. All analyses were performed using SAS statistical software (version 9.4, SAS Institute, Cary NC).

## 3. Results

**Figure 5:**
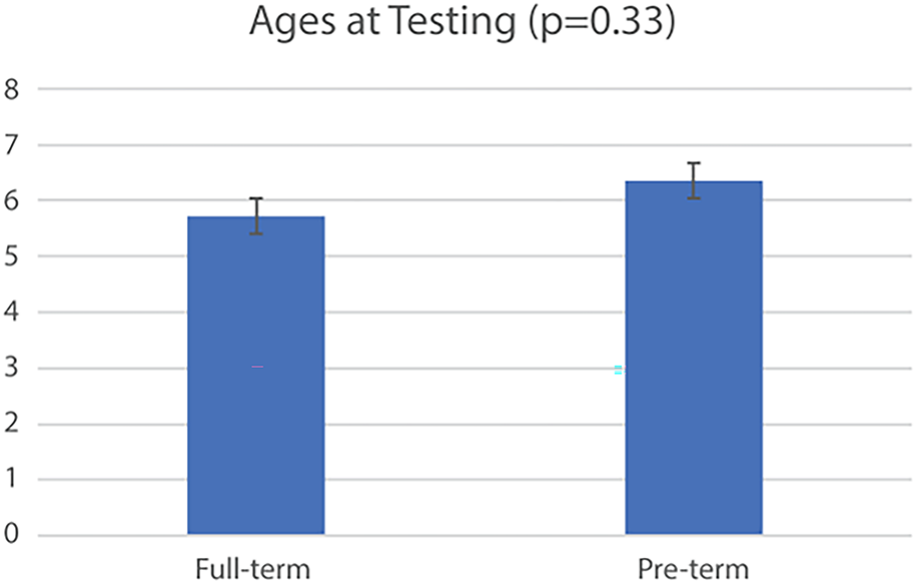
Fourteen infants were born full-term births and nine were pre-term. At testing the average age and standard deviations of each group was Full-term: 5.73 ± 1.47 months and Pre-term: 6.36 ± 1.22 months (*p* = 0.33). In total, we evaluated 19 elephant trials, 18 orangutan trials, and 22 lion trials from 24 infants.

In total, twenty-three infants were included in the current analysis (Table 2), though not all trials from each infant were included. Fourteen were born full-term and nine were pre-term. Individual toy condition trials from these infants were further excluded as necessary (see Figure 1). At testing, the uncorrected average age and standard deviations of each group was Full-term: 5.73 ± 1.47 months and Pre-term: 6.36 ± 1.22 months (*p* = 0.33) (Figure 5). Full median frequency and interquartile ranges of infant engagement with each toy is presented in Table 3.

**Table 2:** Participant characteristics, including health status both at time of testing and 18 months in the future, chronological and corrected age (where applicable), which toys were evaluated for each infant, and motor developmental status at 18 months of age.

Baby #	Health status at testing	Age (months)	Corrected age (months)	Toys evaluated (E, O, L)	Developmental status (@18 months)
6	Full-term	5.0	N/A	E, O, L	Typical
7	Full-term	6.75	N/A	O, L	Typical
8	Full-term	4.5	N/A	E, O, L	Typical
9	Full-term	5.25	N/A	E, O, L	Typical
11	Full-term	4.0	N/A	E, O, L	Typical
14	Full-term	7.5	N/A	L	Typical
17	Full-term	5.25	N/A	E, O, L	Typical
18	Full-term	4.5	N/A	E, O, L	Typical
19	Full-term	6.5	N/A	L	Typical
20	Full-term	4.5	N/A	E, O, L	Typical
21	Full-term	7.25	N/A	E, O, L	Typical
22	Full-term	8.0	N/A	O	Typical
24	Full-term	4.25	N/A	E, O, L	Typical
27	Full-term	7.25	N/A	E, O, L	Typical
15	Pre-term	5.5	2.5	E, O, L	Atypical
16	Pre-term	8.25	5.5	E, O, L	Atypical
28	Pre-term	5.0	2	E, O, L	Atypical
29	Pre-term	7.0	5	E, O, L	Atypical
30	Pre-term	8.0	5	E, O, L	Typical
31	Pre-term	6.5	4	E, O, L	Typical
32	Pre-term	5.0	1.5	E, L	Typical
33	Pre-term	5.5	1	E, L	Atypical
34	Pre-term	6.5	5.5	E, L	Atypical

Data shown for elephant (E), orangutan (O), and lion (L). At testing the average age and standard deviations of each group was Full-term: 5.73 ± 1.47 months and Pre-term: 6.36 ± 1.22 months (*p* = 0.33).

**Table 3:** Median frequency and interquartile range of infant engagement with the toys.

Movement classification frequency and range	Elephant median (IQR)		Orangutan median (IQR)		Lion median (IQR)	
	Full	Pre	Full	Pre	Full	Pre
Involuntary	0.5 (0, 3)	1 (0, 4)	0 (0, 0.5)	1 (0, 4)	3 (1, 10)	0 (0, 0)
Gaze	9.5 (9, 13)	0 (0, 10)	8 (4.5, 10.5)	10.5 (8, 12)	7 (3, 15)	0 (0, 0)
Mouth	0 (0, 2)	0 (0, 0)	1.5 (0, 4.5)	0 (0, 0)	0 (0, 0)	0 (0, 0)
L/R hand touch	12.5 (0, 19)	0 (0, 2)	2.5 (1, 4)	0 (0, 9)	0 (0, 0)	0 (0, 0)
L/R hand grasp	4.5 (0, 11)	0 (0, 0)	5 (2.5, 12)	0 (0, 3)	0 (0, 0)	0 (0, 0)
L/R foot touch	0 (0, 1)	0 (0, 0)	0 (0, 1)	0 (0, 0)	1 (0, 13)	0 (0, 0)
L/R foot kick	0 (0, 0)	0 (0, 0)	0 (0, 0)	0 (0, 0)	0 (0, 3)	0 (0, 0)
Total	22.5 (0, 32)	0 (0, 2)	7 (5, 16)	7 (0, 13)	1 (0, 19)	0 (0, 0)

**Figure 6:**
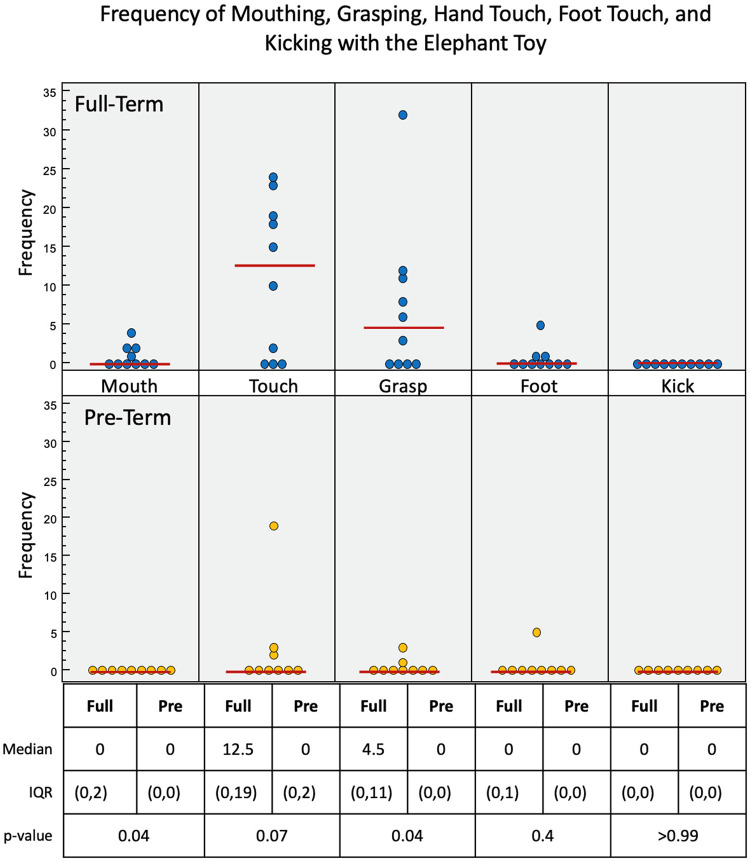
Frequency of infant interactions with the elephant toy. Full-term infant interactions are reported in the top row and pre-term infant interactions in the bottom row. Hand touch and hand grasp show most frequent interactions.

With the elephant toy, upper limb interactions were favored in both groups, with only a few minor foot interactions and no kick interactions recorded for any infant (Figure 6). Full-term infants tended to favor mouth (*p* = 0.04), touch (*p* = 0.07), grasp (*p* = 0.04), and foot (*p* = 0.4) engagement with the toy, with no kick interactions. Full-term infants had a median of 90.7 s of voluntary interaction with the elephant toy, pre-term infants had 0 s (*p* = 0.04).

**Figure 7:**
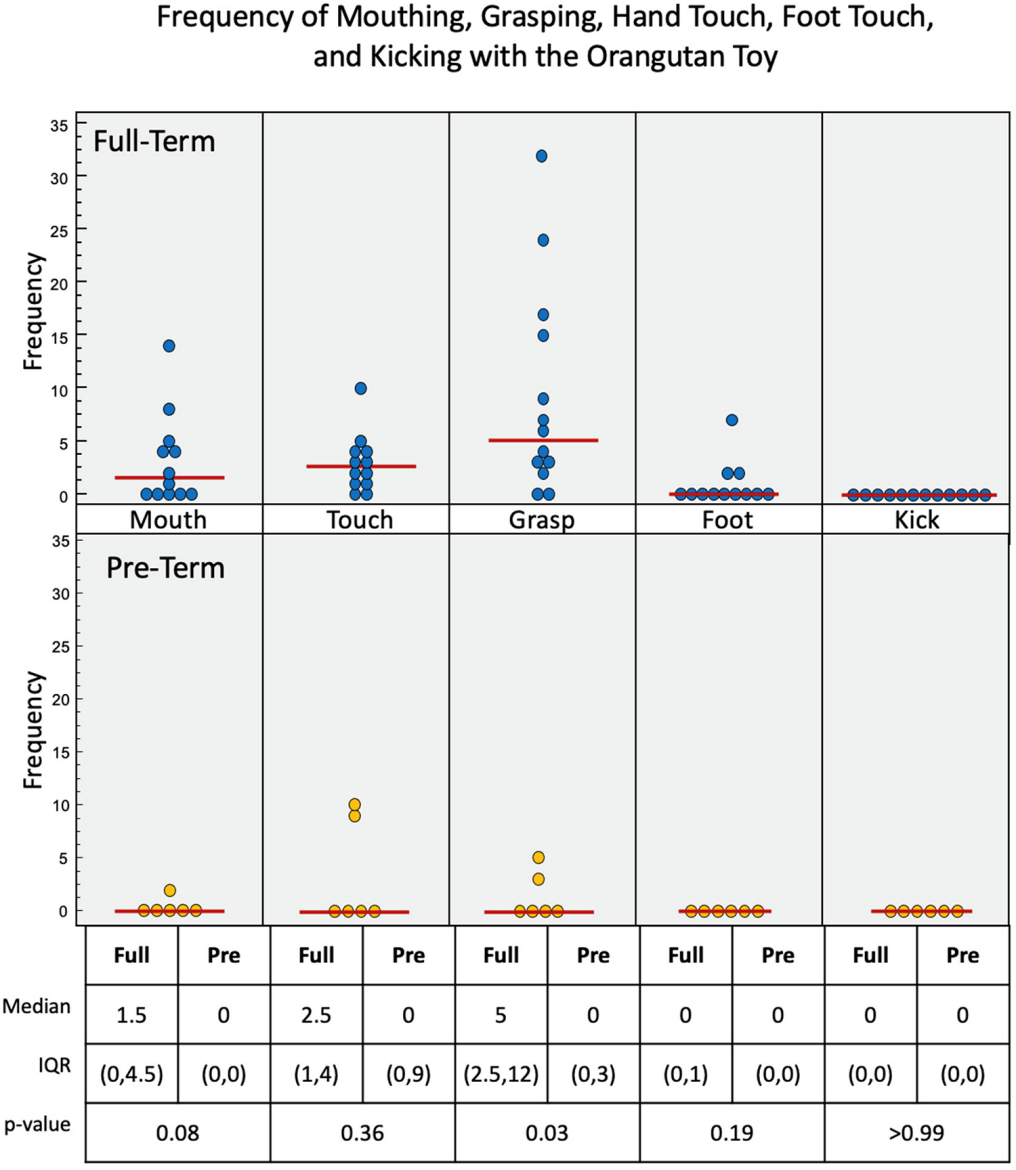
Frequency of infant interactions with the orangutan toy. Full-term infant interactions are reported in the top row and pre-term infant interactions in the bottom row. Mouth, hand touch and hand grasp show most frequent interactions. Some Full-term infants used their feet to interact with this toy.

Upper limb interactions were favored again and observed more often than lower limb interactions with the orangutan toy (Figure 7). There were significant simultaneous interactions, and three of the infants were able to separate the orangutan toy's arms using bimanual interaction. Full-term infants engaged more frequently with the orangutan than pre-term infants. Full-term infants tended to prefer mouth (*p* = 0.08), touch (*p* = 0.36), grasp (*p* = 0.03), and foot (*p* = 0.19) interactions, with no kick interaction. Pre-term infants engaged less frequently overall. But, when they did engage, they utilized mouth, touch, and grasp interaction. Full-term infants had a median of 69.3 s of voluntary interaction with the orangutan toy, pre-term infants had 27.9 s (*p* = 0.22).

**Figure 8:**
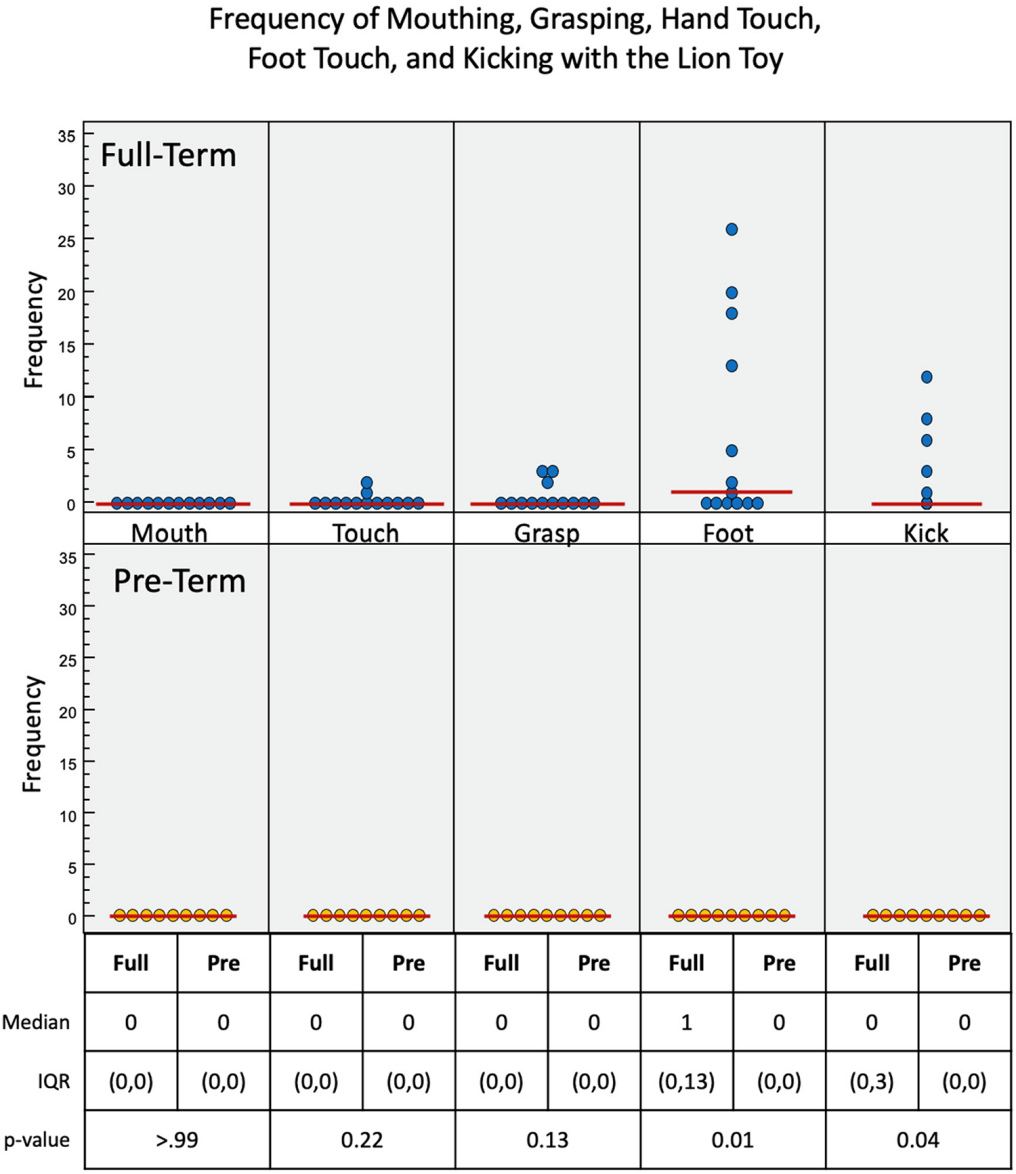
Frequency of infant interactions with the lion toy. Full-term infant interactions are reported in the top row and pre-term infant interactions in the bottom row. Foot and kick show most frequent interactions.

Full-term infants again engaged more with the lion toy (Figure 8), using touch (*p* = 0.22), grasp (*p* = 0.13), foot (*p* = 0.01), and kick (*p* = 0.04) interactions, with no mouth interaction. Pre-term infants had no interaction with the toy at all. Of the three toys used, the lion elicited the least overall interaction. Full-term infants had a median of 2.2 s of voluntary interaction with the orangutan toy, pre-term infants had 0 s (*p* = 0.01).

**Figure 9:**
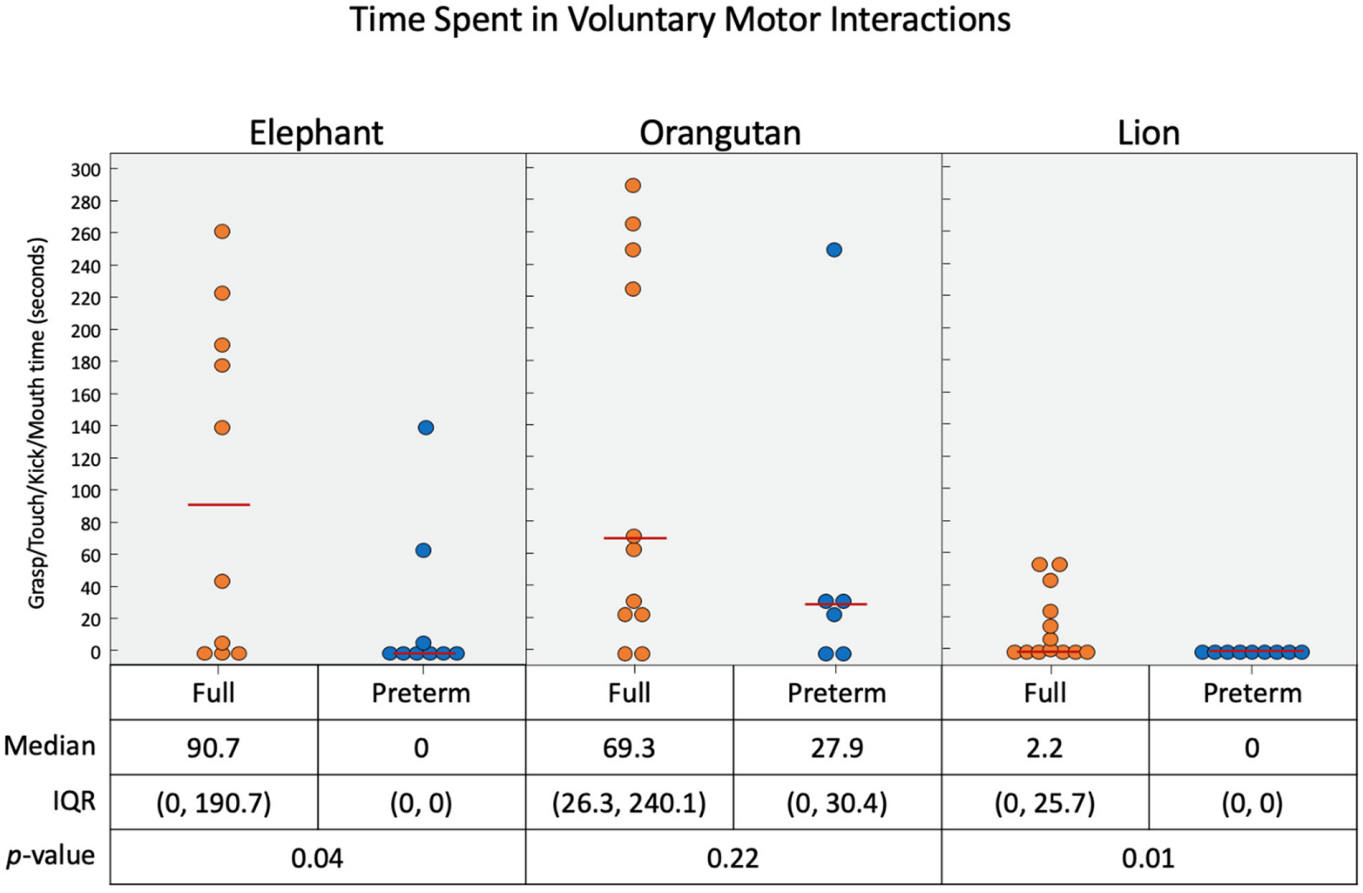
Time duration (in seconds) of infant interaction with the three toys combined. When the voluntary feet and hand interactions are considered in terms of time spent, we see that the elephant and orangutan had most interactions.

As presented in Figure 9, full-term infants engaged for a longer duration with each the elephant, orangutan, and lion toys than the pre-term infants. The full-term infants engaged with each toy to varying degrees, and the pre-term infants engaged with both the elephant and the orangutan but not the lion. The median voluntary movements times (in seconds) for full-term vs. pre-term infants was 90.7 vs. 1.4 for the elephant toy, 69.3 vs. 27.9 for the orangutan toy, and 2.2 vs. 0.0 for the lion toy.

**Figure 10:**
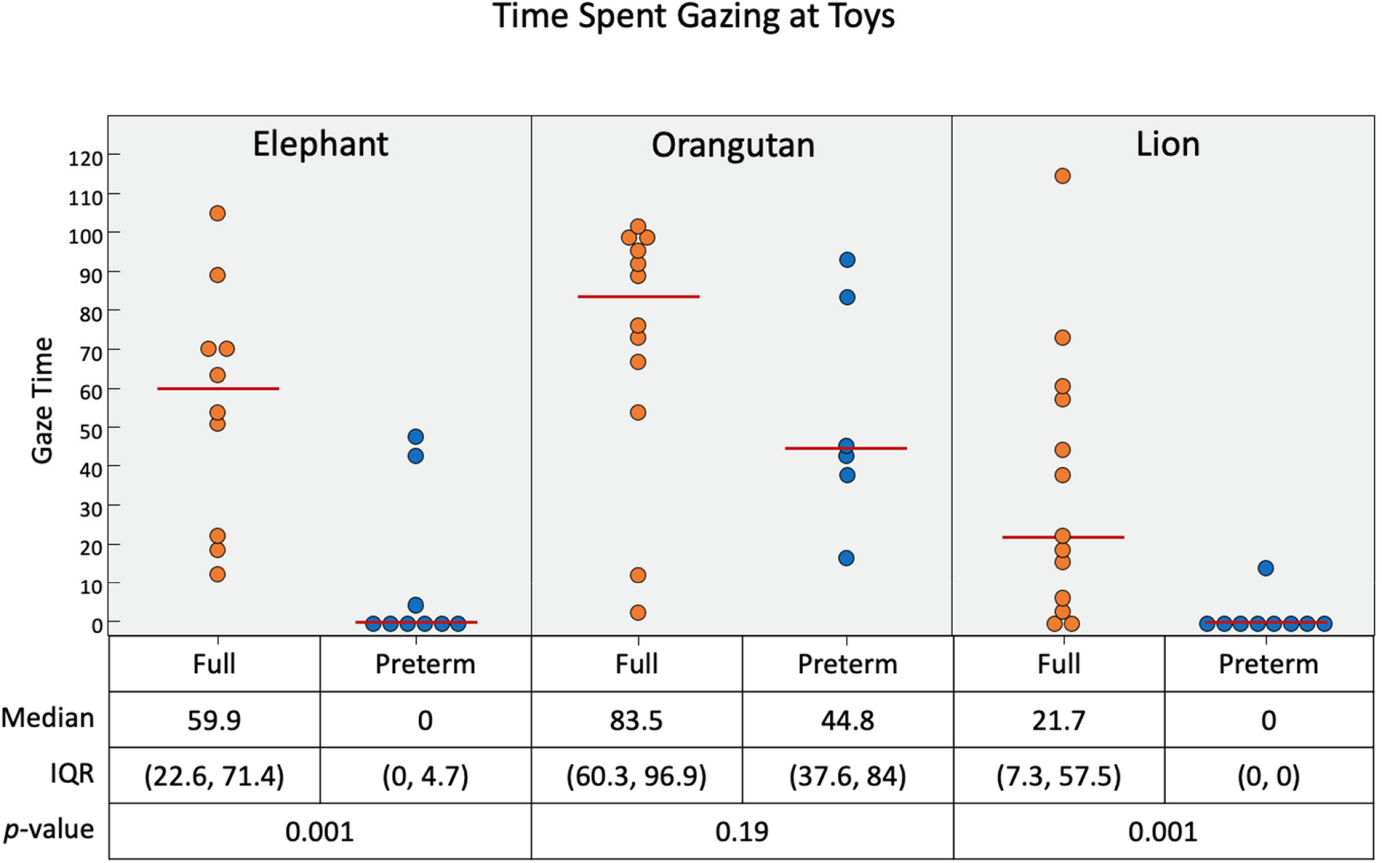
Time duration (in seconds) of infant gaze interaction with the three toys. Pre-term infants were more likely to gaze at the orangutan toy than the elephant and lion. Short black horizontal lines represent median.

As presented in Figure 10, all of the full-term infants engaged with the elephant and the orangutan, and nearly all infants engaged with the lion. Some pre-term infants gazed at each of the toys as well, but most did not. The median gaze times (in seconds) for full-term infants were 59.9 for the elephant toy, 83.5 for the orangutan toy, and 21.7 for the lion toy. Pre-term infants gazed for significantly less time than full-term infants with the elephant toy (*p* = 0.001) and the lion toy (*p* = 0.001) but had similar gaze duration with the orangutan toy (*p* = 0.19).

## 4. Discussion

Our testing paradigm was successful in eliciting visual and motor infant toy interactions and quantifying differences between infants born full term and preterm. As hypothesized, we observed that full-term infants, when compared to pre-term infants, tended to engage more frequently and for a longer duration with each of the toys. All three toys successfully elicited the type of engagement for which they were designed, with the elephant and orangutan toys eliciting more upper limb interactions than the lower limb interactions elicited by the lion.

Results also indicated that gaze and voluntary interactions with legs and arms were heavily influenced by the type of toy and the location of the toys in relation to the infant's supine position. The orangutan toy was the best at eliciting the voluntary arm behaviors and gaze from both infant groups, which was likely due to the toy's positioning at eye-level with the hands hanging near the belly of the infant. The elephant toy was the best at eliciting the voluntary arm behaviors and drawing the gaze of full-term infants. Like the orangutan toy, this was also likely due in part to its positioning at direct eye-level of the infants. The elephant toy's trunk rested near the belly of the infant, which likely also drew attention. Full-term infants more frequently engaged the elephant toy and for longer time durations than all other toys in our trial. The lion toy was less likely to be engaged by any of the infants with respect to frequency, duration, and gaze interaction. Although the lion toy was positioned at the feet of all infants, it did not engage voluntary leg interactions at the frequency and duration expected. However, decreased gaze interaction was expected since the lion toy was positioned further away from many of the infants’ direct eyeline.

Each of the toys elicited some type of gaze interaction from both full- and pre- term infants, but each toy didn't engage physical interaction from both groups equally. While infants in both groups noticed each of the toys and were aware of their presence, not all infants physically engaged with the toys at which they gazed. This primarily occurred in the pre-term infant group and suggests a potential lack of motor skill from infants who otherwise knew a toy was immediately in front of them and, according to expected developmental milestones, should have been able to physically engage.

Pre-term gaze interaction between the two infant groups represents a clear distinction from the expected interaction pattern. While pre-term infants tended to gaze at the toys to a degree that was closer to their full-term counterparts, they were less likely to physically engage with any of the toys. This may suggest that while the pre-term infants were aware of an engaging toy in their immediate space, most of them either did not want to or could not physically interact with it. These results support the Landa et al. (2016) analysis which showed that while both pre-term and full-term infant had looking behaviors, pre-term infants were less likely to follow looking with motor involvement (31).

Our findings support additional work previously seen in the literature. A study by de Soares et al. (2012) which showed that late preterm infants exhibited a greater quantity and proportion of hand engagements and interactions (32). Similarly, a study from Odd et al. (2012) which showed “strong evidence” that later-pre-term infants were at increased risk of developing coordination and cerebral palsy (33). A review from Hadders-Algra (2014) showed that impaired coordination and posture in pre-term infants may contribute to later development of reaching and grasping ability (34).

Hitzert et al. (2015) found that while there was a difference in gaze interactions between pre-term and full-term infants in the first six months extrauterine, this disparity faded over time (35). Gumbsch et al. (2021) researched goal-anticipatory gaze via event-predictive learning and interference and found an event-predictive bias which dictated increased interactions for gaze-coupled interactions (36). Elsner et al. (2021) further found that object-directed grasping (i.e., grasping what they’ve gazed at) to be a skill developed and utilized in the first year of life (37).

Our results show that there may be discernible patterns and mechanisms for identifying and quantifying motor patterns related to age and potentially differences in infant populations who are at risk for motor delay or physical disability. It is possible that quantifying aspects of infant movement and postural control can contribute to the early detection of motor impairment in infants at risk for CP, before motor milestones are missed and allowing earlier initiation of rehabilitation therapies (11). Further, gaze interactions coupled with physical engagement with the toys may be a sensitive and distinguishing feature for neuromotor delay. In a recent study conducted by Landa et al. (2016), both low-risk and high-risk infants demonstrated context-appropriate looking behavior towards an approaching ball, however high-risk infants were less likely to exhibit context-appropriate anticipatory motor response to the ball (moving their arm/hand to intercept the ball) compared to low-risk infants (31). Early characterizations of infant movement remain limited. Beyond the motor and postural requirements for toy interaction, this measurement approach has potential to offer insight on cognitive development as well, including cognitive domains of attention, cause/effect, and motivation to move.

An important limitation of our study is that while we can assert that our tool is sensitive to detect differences in interaction behavior, we cannot assert that we can yet determine if the changes we have discussed and detected are primarily due to differences in development age or a combination. The average uncorrected age of our preterm infants was not significantly different from the average age of our full-term infants, but the corrected ages were and as a result, the groups were not corrected age matched. We do recognize the need to complete further research with a larger sample (Future work) to determine the true source of differences we detected.

There were other factors limiting the study. The sample size of infant subjects was smaller than ideal which may have limited a more robust analysis. Developing and assigning the list of movement interactions was conceptualized from clinician review and thorough literature review, though there was a natural element of qualitative judgement which dictated code assignment. MAXQDA allowed code interactions to be measured to the tenth of a second, which was sufficient for this study's purposes but may hinder other efforts. A more quantitative measure is needed that allows us to automate the detection of the seven interaction types noted in the videos. Further, our manual coding process was time intensive, so an emphasis on future automated methods for coding should be noted. We realized that the use of three robotic toys was not uniquely needed and they could be simplified by combining key features into one general toy as described in our latest toy, Ailu (38).

## 5. Conclusions

We conducted a cross sectional study where the infants were conveniently sampled. The average uncorrected age of our preterm infants was not significantly different from the average age of our full-term infants. Our findings affirm that our tool is sensitive to detect differences in interaction behavior. However, because the groups were not corrected age-matched, we do need to complete further research with a larger sample (future work) to determine if the differences we detected are primarily due to differences in development age or a combination. This future step will be important in determining its utility and predictive validity.

## Data Availability

The original contributions presented in the study are included in the article/Supplementary Material, further inquiries can be directed to the corresponding author.
